# Application of Bladder Acellular Matrix in Urinary Bladder Regeneration: The State of the Art and Future Directions

**DOI:** 10.1155/2015/613439

**Published:** 2015-02-22

**Authors:** Marta Pokrywczynska, Iga Gubanska, Gerard Drewa, Tomasz Drewa

**Affiliations:** ^1^Chair of Regenerative Medicine, Ludwik Rydygier Medical College in Bydgoszcz, Nicolaus Copernicus University in Torun, Karlowicza 24 Street, 85-092 Bydgoszcz, Poland; ^2^Department of Polymer Technology, Gdansk University of Technology, Narutowicza 11/12 Street, 80-233 Gdansk, Poland; ^3^University of Bydgoszcz, Unii Lubelskiej 4C Street, 85-059 Bydgoszcz, Poland; ^4^Department of Urology, Nicolaus Copernicus Hospital, Batory 17-19 Street, 87-100 Torun, Poland

## Abstract

Construction of the urinary bladder *de novo* using tissue engineering technologies is the “holy grail” of reconstructive urology. The search for the ideal biomaterial for urinary bladder reconstruction has been ongoing for decades. One of the most promising biomaterials for this purpose seems to be bladder acellular matrix (BAM). In this review we determine the most important factors, which may affect biological and physical properties of BAM and its regeneration potential in tissue engineered urinary bladder. We also point out the directions in modification of BAM, which include incorporation of exogenous growth factors into the BAM structure. Finally, we discuss the results of the urinary bladder regeneration with cell seeded BAM.

## 1. Introduction

The urinary bladder is a complex organ, whose main functions are storage of urine under low and stable pressure and micturition. There are many clinical conditions, which cause poor bladder compliance and its reduced capacity and require bladder augmentation or substitution. Currently applied surgical procedures utilizing bowel segments are associated with numerous complications, which involve mucus production, chronic bacteriuria, stone formation, ruptures, leakages, fibrosis, electrolyte imbalance, and the development of malignancy at the anastomotic site [1].

The tissue engineering gives the opportunity to construct the urinary bladder wall* de novo* [2, 3]. Numerous natural and synthetic biomaterials have been used for urinary bladder reconstruction with a wide range of outcomes [4]. The most favourable material for urinary bladder reconstruction has to possess good biocompatibility, biodegradation profile, and mechanical properties, especially fatigue strength and elasticity [5]. Due to these requirements, the ideal proposition seems to be extracellular matrix-derived grafts, like bladder acellular matrix (BAM).

BAM is the three-dimensional scaffold of extracellular matrix (ECM) origin. It is composed of typical ECM constituents, which impart to this biomaterial the required biocompatibility and mechanical properties. It also includes growth factors, which regulate the proliferation of cells seeded on the scaffold, stimulate the infiltration of cells from surrounding tissues, and enhance the graft vascularisation [6]. While new functional tissue is being formed, the BAM scaffold undergoes slow degradation, which over time will ultimately result in the remaining presence of just novel tissue (restored in place of implanted scaffold) [7, 8].

In this overview, we focused on bladder acellular matrix preparation techniques and factors that have impact on BAM structure, porosity, and mechanical properties. We also discussed the future directions of BAM developments which are gathered around the incorporation of exogenous growth factors into the BAM structure and BAM seeding with cells. This significantly enhances the regenerative potential of BAM for the urinary bladder. The latest data of published literature indicates that the application of BAM seeded with stem cells will be the main direction of future BAM developments.

## 2. Bladder Acellular Matrix Preparation

Considered pioneers of acellular matrices, Meezan et al. are valuable scientific figures who proposed simple and versatile techniques for biological matrices decellularization and suggested their usage in regenerative medicine [9]. Since that time, there have been several different types of acellular matrices proposed. Some of them like small intestinal submucosa matrices: Surgisis, Durasis, Stratasis (Cook, USA), Oasis (Healthpoint, USA), or acellular dermal matrices: AlloDerm (BioHorizons, USA), Flex HD (Ethicon, USA), DermaMatrix (Synthes, USA), AlloMax (Bard Davol, USA), and SurgiMend (TEI Biosciences, USA), have found wide application in clinical practice [10]. More recently, whole organ decellularization including heart and blood vessel, lung, kidney, liver, and urinary bladder has been proposed as the solution for whole organ tissue engineering [11–16].

Urinary bladder gives the bladder acellular matrix which is successfully used in experimental studies on urinary tract reconstruction. The literature data are full of reports that describe BAM preparation techniques offered over a 40-year period. Many of these proposed techniques were only slightly changed by the authors' modifications [17–21], which shows that they fulfilled their role.

The BAM preparation procedures can be divided into three groups based on how they are applied: (1st) mechanical, (2nd) physical, and (3rd) chemical and/or enzymatic treatments (Table 1). In the 1st group the muscle and mucosal layers are removed mechanically [19, 23, 31–34]. The purpose of these activities is to shorten the time of BAM preparation and the amount of used regents in further steps of BAM preparation (economical reason). The 2nd group is concerned with physical treatments, which involve temperature (freezing/lyophilisation) [35–37] and changes in pressure [38]. This has to cause initial cells lysis, which is further followed by chemical and/or enzymatic treatment (3rd) that is concerned with the use of chemical reagents like sodium azide [19, 22, 23, 31, 32, 39–42], sodium desoxycholate [19, 23, 31, 32, 40–43], hypotonic or hypertonic solutions [17, 21, 44–47], sodium dodecyl sulphate (SDS) [21, 44, 45, 48], Triton X-100 in combination with ammonium hydroxide [27, 28, 30, 34, 35, 49, 50], and DNase or/and RNase [17–19, 21–23, 31, 32, 37, 39–48]. This treatment is more aggressive and is designed to complete bladder decellularization. Table 1 describes the activity of reagents used in decellularization protocols. This way prepared BAM is being subjected to histological and immunohistochemical verification by staining with hematoxylin and eosin (H & E) [19, 22, 23, 30–32, 34–37, 39, 40, 42–45, 48] to indicate the presence of nuclei; trichrome to indicate [19, 23, 30–32, 45] presence of collagen; and antibodies against smooth muscle *α*-actin to indicate [19, 23, 31, 39] presence of smooth muscle. These techniques confirm BAM acellularity.

## 3. Bladder Acellular Matrix Structure, Porosity, and Mechanical Properties

### 3.1. Bladder Acellular Matrix Structure

BAM preparation techniques have been suspected to affect the structure of ECM, which is built from collagen, elastin, fibronectin, laminin, glycosaminoglycans (GAGs), and growth factors. Not only chemical and/or enzymatic treatment but also mechanical procedures which involve mechanical forces influence the BAM structure [51]. The influence of mechanical method used for BAM preparation on its structure was described by Gilbert et al., where they proved that even the delamination direction has the direct impact on BAM mechanical properties [52]. Both structural and mechanical properties of BAM may contribute to the failure of BAM after implantation, due to the fact that BAM has not provided suitable template structure for cell proliferation and vascularisation or does not withstand distension during the urine collection.

Studies comparing the structure of rat, porcine, and human BAM revealed that BAM composition is largely comparable between the species. There are however differences in elastin and collagen fiber types and their abundance between species. Porcines' and humans' BAMs had more elastic fibers than the rats' which are densely packed. Type I collagen is the major component of rats' BAM. On the contrary, type III collagen is abundant in the porcines' and humans' BAM [39]. Chun et al., Farhat et al., and Barnes et al. confirmed the presence of collagen types: I, II, III, and IV, with predominant I and IV type collagen, with less amount of collagen type III in BAM [35, 46, 53]. Badylak reported that collagen type I provides suitable strength for unaxial and multiaxial mechanical loading to which the urinary bladder is constantly subjected [51], and due to its suitable amount found in BAM its mechanical properties are preserved. Elastin, laminin, and fibronectin presence was also confirmed, with little incensement of laminin amount and reduction of fibronectin [35, 46] after urinary bladder decellularization. The preservation of suitable content of laminin is important for BAM applications due to the fact that it stimulates cell binding to collagen type IV and endothelial cell differentiation as well [54]. On the other hand, fibronectin presence in BAM structure is critical for cellular repopulation and proliferation [55]. Due to the fact that all the mentioned collagen types, elastin, laminin, and fibronectin were sustained in BAM structure, this establishes it as an ideal biomaterial for urinary bladder regeneration.

### 3.2. Bladder Acellular Matrix Mechanical Properties

It was reported that the mechanical properties of BAMs were similar for porcines' and humans' BAMs, but significantly greater for rats' BAM (maximum strain, tensile strength, and elastic modulus), the reason being due to the large amount of collagen type I present in the BAM of rat and the lower content of elastic fibers than in the porcines' and humans' BAM [39]. Interestingly, Farhat et al. found out that elastic modulus for prepared porcine BAM was three times higher than for the native one. This was explained by the differences in calculated cross-sectional area of examined acellular samples, which was understandable in case of biological materials [46]. These studies documented that, at the time of implantation, mechanical and structural characteristics of BAM would be maintained [46], but what then would happen with BAM in the prolonged time of implantation? The answer for this question was given by Eberli et al., who performed biomechanical testing on prepared porcine BAMs in long-term, 3-month implantation period. This study indicated that the mechanical properties (such as tensile stress and tensile strain) not change in a significant way in BAMs serving as implants for 3 months. This study then raised another question of possible application of more than one layer of BAM for urinary bladder regeneration, which was suspected to have improved mechanical characteristics [56]. Eberli et al. performed a three-month-time examination of porcine BAMs in the form of a single-layer BAM and a four-layer BAM. Surprisingly, the results indicated that there was no justification in the usage of more than a single-layer BAM, because tensile strain and tensile stress values were similar for each of the materials and were close to the values obtained by the native bladders [57].

No less important studies were done by Freytes et al., who determined porcine BAM mechanical properties after (I) different techniques of decellularization, which were proven to have the direct impact on BAM mechanical characteristics [58], (II) lyophilisation, sterilisation, and storage (up to 12 months), which indicated that structural and mechanical strength changes may be noted in case of long-term storage, but they were not significant [59], and (III) sterilisation procedures—ethylene oxide (EO), gamma irradiation (GI), and electron beam irradiation (e-BI)—which revealed that EO was the most suitable sterilisation technique for BAM to preserve suitable mechanical properties. On the contrary, GI and e-BI directly impacted the mechanical strength of BAM. Moreover, Freytes et al. summarized that the EO is most suitable sterilisation type for materials that would be subjected to low load bearing applications (≪2N) and GI and e-BI for those materials which would have to have improved degradation rate and low mechanical properties [60]. The lyophilisation impact on BAM mechanical properties was also a concern of Feng et al. They found out that there was no significant difference of the Young's modulus and stress at break between lyophilized BAM and BAM prepared at room temperature, but stress at the break was mildly increasing in the case of using lyophilisation [61]. Freytes et al. and Feng et al. studies revealed that the mentioned points: decellularization protocol, sterilisation, and storage, have to be seriously taken into consideration when BAM is being prepared.

### 3.3. Bladder Acellular Matrix Porosity

Porosity is also impacted by decellularization methods. This parameter should also be reflected in the case of the BAM design, due to the fact that too large pores would cause urine leakage which would lead to the chronic bladder inflammation and would result in implant rejection, perforation, and even death [17]. Moreover, too large pore matrix would not provide a suitable environment for cell attachment due to reduced surface and ligand density. Kanematsu et al. compared the porosity of BAMs prepared in two different ways: at room temperature and by lyophilisation. Unlyophilized BAM was water permeable even under low pressure (10 cm H_2_O), while lyophilized BAM had superior waterproofness even after rehydration. The failure point pressure was higher for lyophilized BAMs. Kanematsu et al. studied also the impact of rapid lyophilisation (performed in liquid nitrogen) on BAM porosity, but such BAMs were revealed to be fragile [62]. Feng et al. confirmed that lyophilisation causes loosening of the BAM structure [61]. There are also studies conducted by Eberli et al., which indicated that both single-layer and four-layer BAMs possess great water tightness, which could be useful directly after implantation and affirmed the conviction of application single-layer BAMs over multilayered BAMs [57]. On the other hand, Farhat et al. performed a comparison between porosity of thick (standard decellularization protocol) and thin (modified decellularization protocol) BAM. The results showed that abluminal porosity index (PI) was higher than luminal for both thick and thin BAM. The values of PI were higher in both abluminal and luminal site for the thick BAM than the thin one. More impermeable was the abluminal than the luminal site of thick BAM. A different situation was observed for thin BAM, where the luminal site appeared to be more impermeable than the abluminal one. Luminal site porosity was higher for thin BAM than the thick one. On the contrary, abluminal porosity index was higher for thick BAM than the thin one [17]. However, Cartwright et al. studies showed that BAM porosity can be modified by incorporation of molecules of hyaluronic acid (HA), which has led to decreased BAM porosity [47].

## 4. Bladder Acellular Matrix as Release Carriers of Exogenous Growth Factors

There are two approaches in the usage of growth factors in biomaterials. The first one is related with their endogenous origin concerned with growth factors remaining in naturally derived scaffolds even after the decellularization protocol [63–65]. The second one is related with incorporation of exogenous growth factors in form of their proteins or gene-modified cells expressing growth factors [22, 66–69]. The usage of growth factors is debatable. It is due to the fact that it cannot be exactly concluded if they are uniformly distributed into the BAM structure of each sample and if there is suitable dosage (no more, no less). Moreover, as well as BAM structure, the activity of some endogenous growth factors may be blocked by decellularization protocols (physical changes, like low temperature and lyophilisation process and/or chemical and enzymatic treatments) [63]. The application of exogenous growth factors also did not remain undisputed, due to the fact that too high dosage can be incorporated, which in result would disrupt the suitable healing characteristic or show unexpected events [70]. Despite the fact that the behaviour of growth factors is not fully explained [7, 25], some authors took the challenge to incorporate them into the BAM structure (Table 2) [22–25]. One of the pioneer studies in this area was performed by Kanematsu et al. in 2003. They incorporated into BAM basic fibroblast growth factor (bFGF). The applied technique was very simple. Lyophilized BAM was rehydrated in the bFGF solution. The bFGF releasing profile was formerly studied in mice, before such BAM-carrier construct was implanted into the rat's bladder. The bFGF bioactivity, released* in vivo*, was measured by vascular endothelial growth factor (VEGF) content in the local subcutaneous tissue around the implanted BAM, which reflected the angiogenic activity of bFGF to the endothelium [71]. The release of bFGF was sustained and preserved for a 3-month-time examination. The bladder regeneration was promoted, and what is important is that the graft shrinkage was reduced by 4 weeks postoperatively. However it should be emphasized that there were no differences in the regeneration effect between bladders augmented with BAM and BAM + bFGF at 12 weeks postoperatively [22]. Studies on urethra regeneration proved that exogenous growth factors substantially improved molecular features of healing, but failed to be superior in the functional outcome. Moreover, they indicated that the use of nonoptimal growth factor doses can lead to uncontrolled events such us divericula formation or fibrosis [69].

In another study Kanematsu et al. developed the feasibility of BAM as a growth factor carrier by incorporating bFGF and other factors such as hepatocyte growth factor (HGF), platelet derived growth factor-BB (PDGF-BB), insulin-like growth factor-1 (IGF-1), heparin binding epidermal growth factor-like growth factor (HB-EGF), and VEGF. Results confirmed that sustained release was preserved as follows: bFGF > HGF > PDGF. These growth factors have shown good sustained release profiles, from mouse subcutis, in accordance with the biodegradation of the matrices, which was not always observed in case of HB-EGF, IGF-1, and VEGF [57]. In contrary, Youssif et al. studies carried out in 2005 revealed that VEGF possesses a good release profile and the functional bladder restoration was faster when VEGF was incorporated into the BAM. Additionally important, Youssif et al. found that VEGF enhanced neovascularity and increased smooth muscle content especially in early periods after BAM grafting [23]. Studies of VEGF were also carried out by Loai et al., who evaluated the feasibility of BAM, modified with HA, designed by Cartwright et al. This material had decreased porosity due to the applied modification and it was perfect for growth factor carrier, in which VEGF was incorporated. Loai et al. determined the optimal VEGF dosage, 2 ng VEGF/1 g of tissue for mouse, which is necessary to vascularise implanted BAM and which gives the positive correlation between angiogenesis and fibrosis [24]. The bioactivity of VEGF in correlation with BAM was also evaluated by combining VEGF with other growth factors such as nerve vascular growth factor (NGF) [25] or PDGF-BB [7] or as a gene derivative in form of VEGF-gene modified endothelial progenitor cells (EPCs) [72]. In both cases, such additional modification of BAM with VEGF caused the increase of angiogenesis, neovascularisation, and neurogenesis in reconstructed bladders. There was also enhanced development of smooth muscles and functionality of regenerated bladders. It is important to mention that the growth factors incorporated into BAMs had reduced shrinkage in comparison with native BAMs [7, 25, 72].

Growth factors incorporated into the BAM structure represented one direction of improving BAM functionality. Exogenous growth factors improve bladder regeneration, by enhancement of cell repopulation, and neovascularisation, but due to the fact that they possess short half-lives and have rapid, uncontrolled diffusion, and the optimal doses have not been defined; they are being abandoned in the latest studies in favour of cell studies development.

## 5. Bladder Acellular Matrix as a Scaffold for Cells

Initially, cell-unseeded BAMs were evaluated to be used as urinary bladder implants. In most of these studies the formation of urothelium was observed [44] but accompanied by weak muscular layers, as well as graft shrinkage and scar formation.

It should be emphasized that the environment of the urinary bladder is extremely unsuitable for regeneration due to the toxic effects of urine [73]. The urothelial cells act as a permeability barrier, which protect underlying tissues against urine. Therefore, despite a high proliferative and self-renewal potential of urothelium, seeding of urothelial cells on BAM before implantation seems to be warranted.

First studies were directed to determine favourable conditions of urothelial cell culturing and their cell-cell and cell-BAM interactions [74–76]. Moriya et al. seeded urothelial cells to the BAM surface, which soon after culturing formed a monolayer, and following implantation a multilayer consists of 3-4 cell layers. Unfortunately, the regenerated urothelium was thinner with passing time [77]. Yoo et al. used BAM seeded with urothelial cells and smooth muscle cells for urinary bladder regeneration in a canine model following partial cystectomy. They found that the capacity of the reconstructed bladder increased by 99%. The bladder had completely regenerated urothelium and smooth muscles. In contrast the capacity of bladders augmented with BAM without cells increased only by 30% at 3 months postoperatively [20]. Encouraged by these results Atala et al. used BAM seeded with urothelial cells and smooth muscle cells for the reconstruction of urinary bladders in a pilot human clinical study [78]. The results of this study are questionable because the patients with reconstructed bladders showed deterioration or only a slight improvement in bladder capacity, leak point pressure, and compliance. Some authors in experimental models of urethra regeneration tried to replace the urothelial cells with foreskin epidermal cells or oral keratinocytes as an example, which gave quite satisfactory results, but they now represent the secondary research direction [49, 50].

## 6. Stem Cells and Bladder Acellular in Urinary Bladder Regeneration

Urinary bladder regeneration as described above was initially enhanced by seeding the graft with smooth muscle and urothelial cells [20, 78]. The quite new approach in creation of grafts for urinary bladder augmentation is the usage of stem cells [26–30]. Stem cells can enhance urinary bladder regeneration either directly via differentiation into the urinary bladder cells or indirectly through secretion of growth factors which trigger regeneration. One of the most commonly used stem cell types in urinary bladder reconstruction is mesenchymal stem cells (MSCs). Mesenchymal stem cells (MSCs) were described for the first time in 1976 by Friedenstein et al. [79]. This type of cells was further defined by The International Society of Cellular Therapy as adherent to plastic, expressing specific cell surface antigens (positive expression of CD105, CD73, or CD90 and negative expression of CD45, CD34, CD14, or CD11b, CD79*α*, or CD19 and HLA class II) and able to differentiate into the mesenchymal lineages including osteocytes, adipocytes, and chondrocytes [80, 81]. MSCs can be found in many adult tissues and they are concerned as of the most attractive stem cell type for the regeneration of the damaged tissues [82]. It is due to the fact that they are undifferentiated cells, which are able to self-renew with a high proliferative capacity and possess a multilineage differentiation potential [83]. Bone marrow (BM) has been concerned as a main source of MSCs suitable for isolation, but harvesting process of BM-MSCs is highly invasive. In addition, the number and differentiation potential as well as maximal life span of BM-MSCs decrease with increasing age of cells [84–86]. That is why the alternative sources of stem cells are subjected to constant investigation. Another suggested source of MSCs is adipose tissue. Adipose derived stem cells (ADSCs) may be obtained with less invasive method and in larger quantities than BM-MSCs. It was proved that adipose tissue contains stem cells similar to BM-MSCs. These cells may be simply isolated from cosmetic liposuction in large numbers and grown easily under standard culture conditions [87]. Another attractive source of MSCs is a cord blood (UBC) [88]. Umbilical mesenchymal stem cells (UMSCs) can be obtained with noninvasive methods, which do not cause as much harm as obtaining BM-MSCs. Unfortunately, the number of MSCs in UBC is very low which limits their clinical application [89, 90].

Stem cells isolated from different sources were used to enhance regeneration in reconstructed urinary bladders. The summary of stem cells used for preparation of cell-seeded BAM grafts is presented in Table 3. Zhu et al. evaluated the feasibility of adipose-derived stem cells (ADSCs) seeding into BAM for bladder reconstruction in a rabbit model [27]. Regeneration of smooth muscles, urothelium, and nerves was observed in bladders augmented with ADSCs seeded into BAMs. However, it seems that the nerves and smooth muscles were not organized in comparison to those found in the native bladder wall. However, in bladders augmented with unseeded BAMs, only multilayered urothelium was found. There was no evidence for organized muscles or nerve regeneration. Moreover, the bladders reconstructed with ADSCs seeded BAMs reached 95% of the precystectomy bladder capacity, while bladders reconstructed with unseeded BAMs reached only 70% of the precystectomy volume. This study confirmed that cell seeded BAMs were more suitable for bladder reconstruction than BAM alone [27]. These observations were confirmed by Yuan et al. who evaluated the feasibility of utilizing the human umbilical vein mesenchymal stem cells (HUMSCs) seeded into BAM for urinary bladder reconstruction in the canine model. They also demonstrated the greater regeneration potential of cells seeded compared to unseeded BAM [28].

Pokrywczynska et al. evaluated the influence of bone marrow mesenchymal stem cells (BM-MSCs) seeded onto BAM in urinary bladder regeneration on a rat model. This work indicated that BM-MSCs enhanced smooth muscle regeneration in tissue engineered bladder. Furthermore, they found that BM-MSCs modulated the milieu of the reconstructed bladder wall by upregulation of the expression of anti-inflammatory cytokines, which probably enhanced the regeneration process [29].

## 7. Conclusions

In this overview we exposed the potential of BAM application for urinary bladder regeneration. BAM, due to its natural origin, possesses favourable morphology. Such suitable composition as found in BAM provides the possibility of it to function as a stable template for cell ingrowth into the scaffold, as well as channels for nutrients needed for cell proliferation and differentiation. Remaining growth factors cannot be underscored in their function, and preservation of unaltered mechanical properties and porosity are significant. Mentioned physicochemical and mechanical properties of BAM create the strong fundament for further developments in this field, which current published data supports, direct to the BAM seeding with cells, mostly mesenchymal stem cells, as a suitable construct for bladder regeneration, which give satisfactory results and get the studies closer to the applied tissue engineering of the urinary bladder.

## Figures and Tables

**Table 1:** Agents used in urinary bladder acellular matrix preparation.

Treatment		Main role	Example	Advantages	Disadvantages
Mechanical	Force	Removal of tissue layers	Scrapping with the use of the scalpel	Some tissues like bladder and intestinal submucosa have natural planes of dissection and therefore mechanical force can be used to delaminate the tissue layers	Not all tissues can be mechanically treated, because it can disturb the ECM structure

Physical	Temperature	Cell membrane lysis	Freezing cycles	Effective when combined with full decellularization protocol	Applied alone, would not remove cells completely
	Pressure		Hydrostatic pressure	Effective for tissues which do not have densely organized ECM, such as liver and lungs	Used alone, would not completely remove the cells

Chemical	Acids and alkalis	Protein denaturation, solubilisation of cells elements, initial nucleic acid infraction	Acetic acid, peracetic acid, hydrochloric acid, sulphuric acid, paracetic acid ammonium hydroxide, sodium azide, sodium deoxycholate	Effective	Not selective, possible alternation of ECM constituents: collagen, GAG, growth factors
	Hypertonic/hypotonic solutions	Cell disruption by the osmotic shock, disruption of DNA-protein interaction	Tris/HCl	Efficient	Do not effectively remove the cellular residues
	Ionic/nonionic detergents	Destroy DNA-protein interactions, lipids, and lipoproteins	Triton X-100 SDS	Effective, destroy lipid-lipid and lipid-protein interaction but keep protein-protein interactions	Possible protein denaturation, loss of GAGs, laminin, and fibronectin
	solvents	Dehydratation cells lysis lipids removal	Alcohol, glycerol, acetone	Effective	Possible ECM constituent destruction

Enzymatic	Enzymes	Cells rupture destruction of peptide bonds destruction of nucleic acids	Trypsin, DNase/RNase	Targets the residues of nucleic acids	May remain in the tissue with unknown amount and intensify the immune response of the host tissues

ECM: extracellular matrix; SDS: sodium dodecyl sulphate; GAGs: glycosaminoglycans.

**Table 2:** Growth factors incorporated with bladder acellular matrix and their effects on urinary bladder regeneration.

Author, year [Reference no.]	Growth factor	Growth factor incorporation technique	Experimental model	Urinary bladder regeneration	
				BAM	BAM + exogenous growth factors
Kanematsu et al. 2003 [22]	bFGF	Reswelling of the freeze-dried or not-freeze-dried BAM in PBS containing the bFGF radiolabeled with Na^125^I by chloramine T.	Rat	(i) Graft shrinkage, (ii) no differences in capacity, compliance, and max pressure between bladders augmented with BAM and BAM + bFGF, (iii) complete regeneration of mucosal and smooth muscle layers at 12 weeks postoperatively (no differences between BAM and BAM + bFGF groups)	(i) Graft shrinkage was reduced (bFGF dose dependent manner) in BAM + bFGF compared to BAM group at 4 weeks postoperatively, (ii) no differences in graft shrinkage between BAM and BAM + bFGF groups at 12 weeks postoperatively, (iii) increased angiogenesis (bFGF dose dependent manner) in BAM + bFGF compared to BAM group

Youssif et al. 2005 [23]	VEGF	Incubation of BAM for 12 hours at 37°C in solution containing VEGF, additionally before implantation of the VEGF solution was injected into the 4 areas of the BAM	Rat	(i) No significant differences in bladder capacity, compliance, and intravesical pressure at 8 weeks postoperatively between BAM and BAM + VEGF groups, (ii) completely regenerated urothelium at 2 weeks postoperatively (no differences between BAM and BAM + VEGF groups)	(i) Increased neovascularity in BAM + VEGF group compared to BAM group at all points of observation (2, 4, 8, and 12 weeks), (ii) higher smooth muscle content in BAM + VEGF group compared to BAM group at all points of observation, (iii) increased level of nerve fibers at 2, 4, and 8 weeks, but comparable at 12 weeks in the BAM + VEGF group compared to BAM group

Loai et al. 2010 [24]	VEGF	Lyophilized BAM was rehydrated in HA, dehydrated in ethanol, lyophilized for the second time, and then rehydrated in VEGF solution	Pig	(i) Poor organization of smooth muscle fibers in peripheral and central areas of the graft in BAM-HA and BAM groups at 10 weeks postoperatively, (ii) lower expression of UPIII in urothelium of bladders augmented with BAM compared to BAM-HA-VEGF and BAM-HA	(i) Increased recellularization in BAM-HA-VEGF group compared to BAM-HA and BAM groups, (ii) increased expression of angiogenic markers: CD31 and factor VIII in BAM-HA-VEGF group compared to BAM group, (iii) well organized smooth muscles bundles comparable to native bladder in BAM-HA-VEGF group at 10 weeks postoperatively

Kikuno et al. 2009 [43]	NGF and VEGF	NGF and VEGF were injected into the 4 points of the bladder submucosa	Rat	(i) Urothelium covered completely the luminal surface of implanted BAM in all groups, (ii) well defined smooth muscle layer was observed in all groups	(i) Bladder capacity and compliance were much higher in BAM + NGF + VEGF group compared with BAM + NGF, BAM + VEGF or BAM groups, (ii) smooth muscle content and number of PGP 9.5 nerve fibers were significantly higher in BAM + NGF + VEGF compared to other groups

BAM, bladder acellular matrix; bFGF, basic fibroblast growth factor; HA, hyaluronic acid; NGF, nerve growth factor, PGP 9.5, protein gene product 9.5; PBS, phosphate buffered saline; VEGF, vascular endothelial growth factor.

**Table 3:** Stem cell types seeded on bladder acellular matrix and their effects on urinary bladder regeneration.

Author, year [Reference no.]	Cells type	Experimental model	Urinary bladder regeneration	
			BAM seeded with cells	Unseeded BAM
Drewa et al. 2009 [26]	Hair follicle stem cells	Rat	(i) All animals survived the observation period, (ii) stone disease in one animal (11%), (iii) regular shape of reconstructed bladders confirmed by cystography, (iv) smooth muscle regrowth similar to the normal pattern of detrusor muscle, (v) a structure resembling a hair follicle in the bladder of one animal (11%)	(i) Unexpected deaths due to the leakage and pyuria in two animals (22%), (ii) stone disease in six animals (67%), (iii) irregular shape of reconstructed bladders confirmed by cystography, (iv) extremely thin smooth muscle layer in the central parts of reconstructed area, (v) a thin urothelial layer

Zhu et al. 2010 [27]	Adipose derived stem cells	Rabbit	(i) All animals survived the observation period, (ii) lack of graft shrinkage, (iii) 95% of the original bladder capacity at 24 weeks postoperatively, (iv) multilayered urothelium at implanted site at 4 weeks postoperatively, (v) appearance of native bladder wall at 24 weeks postoperatively, (vi) organized smooth muscle tissue, neoangiogenesis, and proliferation of neural cells	(i) One animal (8%) died due to infection secondary to bladder leakage, (ii) graft shrinkage, (iii) 69% of the original bladder capacity at 24 weeks postoperatively, (iv) multilayered urothelium at implanted site at 4 weeks postoperatively, (v) the smooth muscle cells at the periphery of the graft organized but distinguishable from normal bladder tissue

Yuan et al. 2013 [28]	Human umbilical mesenchymal stem cells	Dog	(i) All animals survived the observation period, (ii) grafts covered by soft, vascularised, connective tissue (iii) no stones, tumours, diverticulum formations, (iv) complete regenerated urothelium and well developed smooth muscles at 12 weeks postoperatively	(i) All animals survived the observation period, (ii) grafts covered by soft, vascularised, connective tissue (iii) no stones, tumours, diverticulum formations (iv) multilayered urothelium and weekly developed smooth muscles at 12 weeks postoperatively

Pokrywczynska et al. 2013 [29]	Bone marrow mesenchymal stem cells	Rat	(i) All animals survived the observation period, (ii) lack of graft shrinkage, (iii) irregularly distributed smooth muscle fibres, (iv) 61% of native smooth muscle content 12 weeks postoperatively, (v) hyperplasic urothelium observed in three cases (60%), (vi) increased capillary density, (vii) presence of nerves in reconstructed area, (viii) increased expression of anti-inflammatory cytokines in tissue engineered bladder wall	(i) All animals survived the observation period, (ii) graft shrinkage, (iii) extremely thin or complete absence of smooth muscle layer, (iv) 25% of native smooth muscle content 12 weeks postoperatively, (v) hyperplasic urothelium observed in all cases (100%), (vi) lack of nerves in reconstructed area

Leite et al. 2014 [30]	Bone marrow mesenchymal stem cells	Rat	(i) No reduction of inflammation over time (7–28 days after bladder augmentation), (ii) architecture of the muscular layer similar to native bladder at 28 days postoperatively, (iii) absence of well formed neural network	(i) No reduction of inflammation over time (7–28 days after bladder augmentation), (ii) disorganized architecture of the muscular layer, at 28 days postoperatively, (iii) absence of well formed neural network

BAM: bladder acellular matrix.
